# MicroRNA-126 inhibits colon cancer cell proliferation and invasion by targeting the chemokine (C-X-C motif) receptor 4 and Ras homolog gene family, member A, signaling pathway

**DOI:** 10.18632/oncotarget.11176

**Published:** 2016-08-10

**Authors:** Wei Yuan, Ye-Qing Guo, Xia-Yu Li, Min-Zi Deng, Zhao-Hua Shen, Chi-Bin Bo, Ya-Fei Dai, Ming-Yu Huang, Zhen-Yu Yang, Yong-Sheng Quan, Li Tian, Xiaoyan Wang

**Affiliations:** ^1^ Department of Gastroenterology, The Third Xiangya Hospital of Central South University, Changsha, Hunan, China; ^2^ Hunan Key Laboratory of Nonresolving Inflammation and Cancer, Changsha, Hunan, China; ^3^ Department of Gastroenterology, Yichang Central People's Hospital, Yichang, Hubei, China; ^4^ Cancer Research Institute, Central South University, Key Laboratory of Carcinogenesis, Ministry of Health, Key Laboratory of Carcinogenesis and Cancer Invasion, Ministry of Education, Changsha, Hunan, China

**Keywords:** colon cancer, MicroRNA-126 (miR-126), CXCR4, RhoA

## Abstract

MicroRNA-126 (miR-126) suppresses the migration, proliferation and invasion of colon cancer cells. However, the underlying mechanisms of miR-126 in colon cancer have not been fully elucidated. In this study, *in vivo* experiments revealed that miR-126 inhibits colon cancer growth and metastasis. Furthermore, miR-126 was down-regulated in human colon cancer tissue, and its expression was inversely correlated with TNM stage and metastasis of patients. Low level of miR-126 identified patients with poor prognosis. And we found that miR-126 expression was negatively correlated with the expression levels of chemokine (C-X-C motif) receptor 4 (CXCR4) and components of signaling pathway of Ras homolog gene family, member A (RhoA) *in vitro* and *in vivo*. Moreover, we verified that miR-126 negatively regulated CXCR4 and RhoA signaling *in vitro*. In addition, either in miR-126-overexpressing or in miR- 126-silenced colon cancer cells, the restoration of CXCR4 could significantly reverse the proliferation and invasion, as well as abolish the effects of miR-126 on RhoA signaling pathway. Collectively, these results demonstrated that miR-126 acts as a tumor suppressor by inactivating RhoA signaling via CXCR4 in colon cancer. And miR-126 may serve as a prognostic marker for monitoring and treating colon cancer.

## INTRODUCTION

Colon cancer is a major cause of cancer-associated deaths worldwide [1]. Despite great improvements in its diagnosis and treatment, invasion and metastasis of colon cancer cells to proximal and distant organs remains the major treatment challenge. Thus, it is important to elucidate the molecular mechanisms of colon cancer progression, especially those with metastasis, to improve diagnosis and treatment efficacy.

MicroRNAs (miRNAs) are small non-coding RNAs consisting of 20–25 nucleotides [2] that regulate gene expression by binding to the 3′-untranslated regions of their target mRNAs, resulting in the degradation or translational repression [3]. Emerging evidence shows that many different miRNAs are aberrantly expressed in various cancer cells, and associated with tumor initiation, promotion, and progression via their interaction with oncogenes and tumor suppressor genes [4–6].

MiR-126 is located at chromosome 9q34.3 within intron 7 of the epidermal growth factor-like domain 7 gene and acts as a tumor suppressive miRNA in various solid tumors, including human gastric cancer [7, 8], where its down-regulation facilitates angiogenesis via the up- regulation of VEGF-A [7]. In addition, restoration of miR-126 expression *in vitro* and *in vivo* down-regulates VEGF-A expression and thereby inhibits lung cancer cell proliferation [9]. MiR-126 also inhibits the proliferation of small cell lung cancer cells by targeting solute carrier family 7, member 5 [10]. Consistent with them, we previously demonstrated that miR-126 acted as a tumor suppressor in colon cancer *in vitro,* and it suppressed colon cancer cell proliferation, invasion and migration [11–13].

Chemokine (C-X-C motif) receptor 4 (CXCR4), a member of the seven-transmembrane G-protein-coupled receptors, is crucial for the mobilization, migration, proliferation, and survival of many cell types [14–17]. CXCR4 is highly expressed in various cancer types and is considered as the most widely expressed cancer-associated chemokine receptor [18–23]. Consistent with the aforementioned observations, we previously verified that CXCR4 is a target for miR-126-mediated repression, and found that this repression inhibits colon cancer cell migration and invasion [11].

The Ras homolog gene family, member A (RhoA) is the most extensively studied member of the Rho GTPase family [24]. RhoA is associated with invasion and poor prognosis in colorectal cancer [25]. Rho exerts its functions through downstream Rho effectors such as PI3K (phosphatidylinositol 3-kinase) [26], ROCK (Rho-associated coiled coil forming protein kinase) [27], PAK (p21-activated kinase) [28] and PKN (protein kinase C-related kinase) [27].

The Rho guanine nucleotide exchange factors (RhoGEFs) activate Rho GTPase, while Rho GTPase-Activating Proteins (RhoGAPs) (including RhoGTPase activating proteins 5 (ARHGAP5) can negatively regulate Rho GTPase. We previously found that miR-126 acts as tumor suppressor via RhoA/ROCK inhibition in colon cancer cells [12], but the precise roles of miR-126 in colon cancer and the underlying mechanisms remain unclear.

In this study, we present evidence that reduction in miR-126 expression, up-regulation of CXCR4 and components of the RhoA signaling pathway in colon cancer tissues were significantly correlated with TNM stages, lymph node metastasis and poor clinical outcome. *In vivo* and *in vitro*, miR-126 suppressed the invasion and migration of the colon cancer cells by down-regulating CXCR4 and inactivating the RhoA signaling pathway. In addition, either in miR-126-overexpressing or in miR-126-silenced colon cancer cells, the restoration of CXCR4 could significantly reverse miR-126-induced suppression of colon cancer cell migration, invasion and proliferation, as well as abolish the effects of miR-126 on RhoA signaling. Taken together, our findings suggested that miR-126 acts as a tumor suppressor by inactivating the RhoA signaling pathway via CXCR4 in colon cancer. And miR-126 may serve as a prognostic marker for monitoring and treating colon cancer.

## RESULTS

### MiR-126 inhibits tumorigenicity and metastasis *in vivo*

Our previous studies showed that miR-126 suppressed migration, proliferation and invasion of colon cancer cells *in vitro* [11, 12]. In this study, we then assessed the effects of miR-126 on tumorigenicity and metastasis *in vivo*. To this end, stable miR-126 over-expression in HCT116 cells and miR-126 suppression in SW480 cells were established using lentivirus-based delivery. The expression of miR-126 in these stable transfected cells was verified by quantitative reverse transcription-PCR (qRT-PCR; Supplementary Figure S1). The *in vivo* roles of miR-126 in cell growth and migration were then assessed through tumour formation following subcutaneous or intravenous injection into nude mice with colon cancer cells that had miR-126 either stably over-expressed or suppressed.

**Figure 1:**
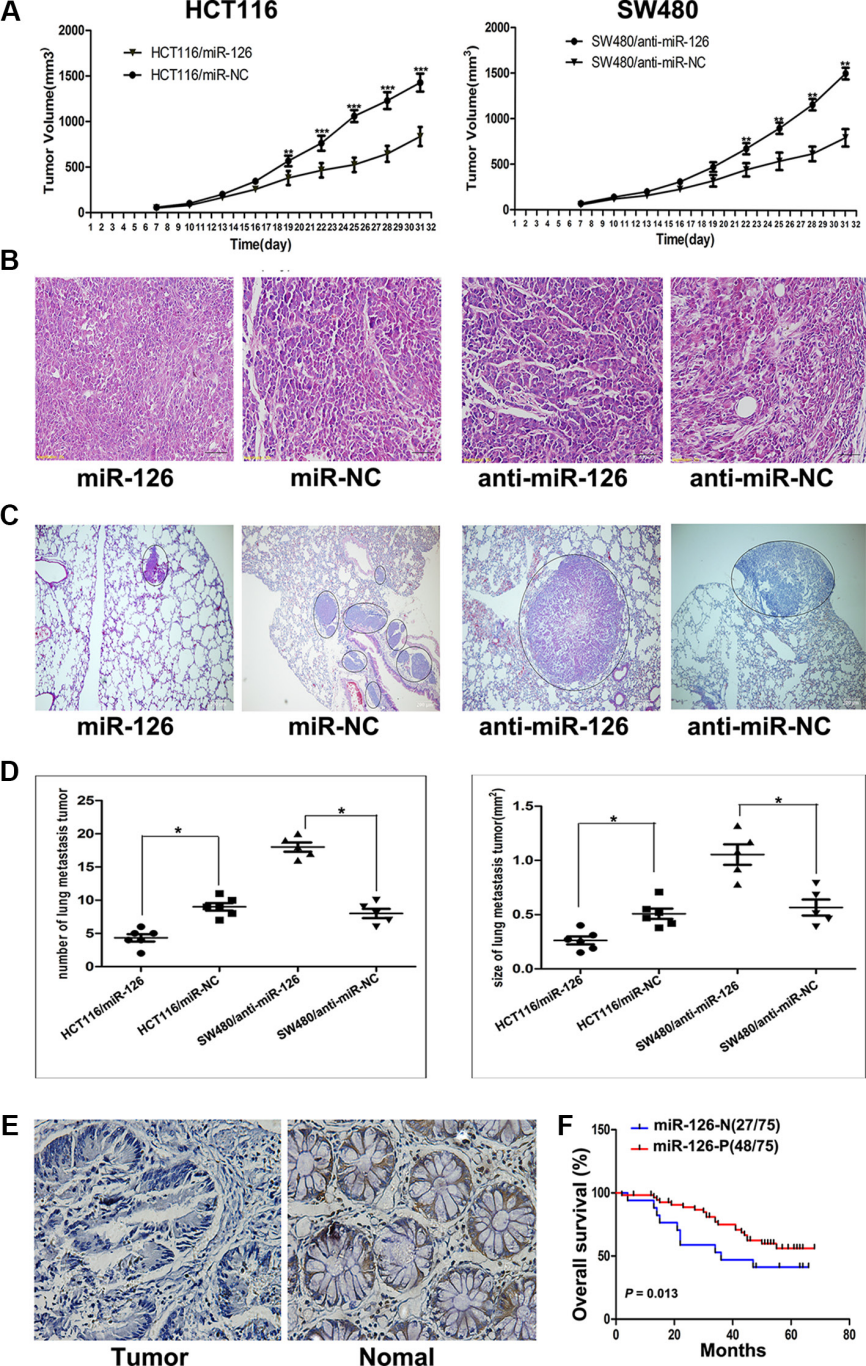
miR-126 inhibits colon cancer progression (**A**) Tumor volume was measured 1 week after injection and every 3 d thereafter. The average tumor volume for HCT116/miR-126 was smaller than that of the control (HCT116/miR-NC; left panel; *n* = 5, *p* < 0.01, ANOVA), whereas the average tumor volume for SW480/anti-miR-126 was larger than that of the control (SW480/anti-miR-NC; right panel; *n* = 5, *p* < 0.01, ANOVA), **p < 0.01, ***p < 0.001. (**B**) Representative hematoxylin/eosin-stained images of subcutaneous tumors from mice that had been injected with transfected HCT116 or transfected SW480 cells (200×). (**C**) Representative hematoxylin/eosin-stained images of lung tissue sections from mice that had been injected with transfected HCT116 (left panel) or transfected SW480 cells (right panel). Black circles identify metastatic foci (200×). (**D**) Scatter diagrams depicting the average number (left panel) and size (right panel) of metastatic nodules in 4–5 microscope fields for each type of transfected cells, *p < 0.05. (**E**) A decrease in miR-126 was detected in microarrays of colon cancer tissues compared with that detected in normal colonic mucosa as shown by *in situ* hybridization (200×). (**F**) Kaplan-Meier survival curves for patients with different levels of miR-126 expression in their colon tumors. Red line: patients whose tissues expressed miR-126 (48/75 patients); blue line, patients whose tissues did not express miR-126 (27/75 patients). *p* = 0.013. MiR-126-N: miR-126 negative expression; miR-126-P: miR-126 positive expression.

Tumorigenicity assay revealed that the nude mice injected with miR-126-overexpressing HCT116 cells formed smaller subcutaneous tumors than those injected with the control cells (*n* = 6/group; *p* < 0.01, ANOVA, Figure 1A, left panel). And the nude mice injected with miR-126-silenced SW480 cells had larger subcutaneous tumors compared with those injected with the control cells (*n* = 6/group; *p* < 0.01, ANOVA, Figure 1A, right panel). The presence of subcutaneous tumors was examined by hematoxylin/eosin staining 31 d after subcutaneous injection (Figure 1B).

For the metastasis assay, the mice were sacrificed 50 d after intravenous injection, and the metastatic nodules in the lung that were derived from the colon cancer cells were evaluated (Figure 1C). The number and area of the lung metastatic nodules were significantly decreased in the mice injected with miR-126-overexpressing HCT116 cells compared with those injected with the control cells (*n* = 5–6/group; *p* < 0.05, ANOVA), whereas a significantly increased number and area of lung metastatic nodules were observed in mice injected with miR-126-silenced SW480 cells compared with those injected with the control cells (*n* = 5–6/group; *p* < 0.05, ANOVA, Figure 1D). Collectively, these results showed that miR-126 inhibits the tumorigenicity and metastasis of colon cancer cells *in vivo*.

### MiR-126 expression is down-regulated in colon cancer tissues and inversely correlated with clinical stage, lymph node metastasis and overall survival of colon cancer patients

Our previous and present studies revealed that miR-126 inhibits colon cancer progression *in vitro* and *in vivo*. To extend our observations to human colon cancer tissues, *in situ* hybridization (ISH) of miR-126 was performed using microarrays of human colon cancer and normal colon mucosa tissues from 75 individuals. The *in situ* hybridization assay revealed that the expression of miR-126 was significantly down-regulated in the colon cancer tissues compared to normal colon mucosa tissues (*p* < 0.01, Figure 1E).

We next characterized the relationship between miR- 126 expression levels and clinicopathologic characteristics of the colon cancer patients who had donated the microarray tissues. The relationship between the miR-126 expression levels and the clinicopathological characteristics of colon cancer patients are summarized in Table 1. Our results revealed that miR- 126 level was negatively associated with TNM stage (*p* < 0.05) and lymph node metastasis (*p* < 0.05) but not with age, gender, tumor size or tumor differentiation. We conducted a 5-year follow-up of the patients and constructed Kaplan-Meier plots to determine the relationship between overall survival time and miR-126 level (*n* = 75). We found that the 5-year overall survival rate of the patients with the negative miR- 126 expression was lower than that of the positive miR-126 expression group (Figure 1F; *p* = 0.013).

**Table 1:** Relationships between the expression of miR-126, CXCR4, and RhoA signaling pathway components and colon cancer clinicopathological features

Expression	MiR-126 (%)	CXCR4 (%)	RhoA (%)	RhoGEF (%)	ARHGAP5 (%)	PI3K (%)	ROCK (%)	PAK (%)	PKN (%)
**Location**									
Colon cancer	48/75 (64.0)	50/66 (75.7)	47/69 (68.1)	56/67 (83.5)	55/64 (85.9)	46/64 (71.8)	48/69 (69.6)	47/66 (71.2)	58/69 (84.1)
Healthy mucosa	54/59 (91.5)	36/64 (56.2)	30/67 (44.7)	43/63 (68.3)	33/63 (52.3)	35/68 (51.5)	27/64 (42.2)	28/67 (41.8)	33/66 (50.0)
*p*	*P*< 0.001	0.019	0.006	0.040	*P*< 0.001	0.016	0.001	0.001	*P*< 0.001
**Age**									
≥ 60	32/49 (65.3)	29/40 (72.5)	33/47 (70.2)	37/45 (88.2)	33/40 (82.5)	31/44 (70.4)	33/45 (73.3)	32/42 (76.2)	33/40 (82.5)
< 60	16/26 (61.5)	21/26 (80.7)	14/22 (63.6)	19/22 (86.4)	22/24 (91.7)	12/20 (60.0)	15/24 (62.5)	15/24 (62.5)	25/29 (86.2)
*p*	0.746	0.444	0.585	0.937	0.516	0.210	0.352	0.237	0.935
**Gender**									
Male	22/38 (57.9)	26/35 (74.3)	26/35 (74.3)	30/37 (81.1)	31/36 (88.5)	28/38 (73.7)	27/40 (67.5)	26/37 (70.2)	32/38 (84.2)
Female	26/37 (70.3)	24/31 (77.4)	21/34 (61.8)	26/30 (86.7)	24/28 (85.7)	18/26 (69.2)	21/29 (72.4)	21/29 (72.4)	26/31 (83.9)
*p*	0.264	0.767	0.265	0.778	1.000	0.697	0.661	0.849	1.000
**Differentiation**									
Well	43/63(68.3)	42/54 (77.8)	39/57 (68.4)	46/55 (83.6)	43/52 (82.6)12/12	36/52 (69.2)	40/57 (70.2)	37/54 (68.5)	49/60 (81.7)
Poor	5/12 (41.7)	8/12 (66.7)	8/12 (66.7)	10/12 (83.3)	(100.0)	10/12 (83.3)	8/12 (66.7)	10/12 (83.3)	9/9 (100.0)
*p*	0.153	0.660	0.906	1.000	0.274	0.533	0.810	0.305	0.361
**TNM stage**									
I–II	27/35 (77.1)	20/32 (66.7)	20/36 (55.6)	24/33 (96.9)	26/32 (81.3)	20/33 (60.6)	17/33 (51.5)	22/33 (66.7)	27/33 (81.8)
III–IV	21/40 (52.5)	30/34 (88.2)	27/33 (81.8)	32/34 (94.1)	29/32 (90.6)	26/31 (83.9)	31/36 (86.1)	25/33 (75.8)	31/36 (86.1)
*p*	0.027	0.015	0.019	0.018	0.472	0.039	0.002	0.415	0.627
**Metastasis**									
Yes	19/37 (51.4)	29/33 (87.9)	29/34 (85.3)	25/34 (64.7)	29/35 (82.9)	19/33 (57.5)	16/33 (48.5)	24/36 (66.7)	27/34 (73.5)
No	29/38 (76.3)	21/33 (63.6)	18/35 (51.4)	31/33 (93.9)	26/29 (89.7)	27/31 (87.1)	32/36 (88.9)	23/30 (76.7)	31/35 (94.2)
*p*	0.024	0.022	0.003	0.024	0.676	0.009	0.001	0.372	0.299
**Tumor size**									
≥ 5 cm	26/41 (78.1)	29/39 (82.1)	26/40 (67.5)	31/39 (79.5)	30/34 (88.2)	26/37 (82.1)	29/42 (69.0)	27/38 (71.0)	36/42 (85.7)
< 5 cm	22/34 (82.4)	21/27 (85.1)	21/29 (75.8)	25/28 (89.3)	25/30 (83.3)	20/27 (85.1)	19/27 (70.4)	20/28 (71.4)	22/27 (81.5)
*p*	0.908	0.750	0.514	0.463	0.839	0.738	0.907	0.973	0.895

### CXCR4 and components of the RhoA signaling pathway are significantly up-regulated in colon cancer, and their expression levels positively correlate with clinical stage, lymph node metastasis and overall survival of colon cancer patients

**Figure 2:**
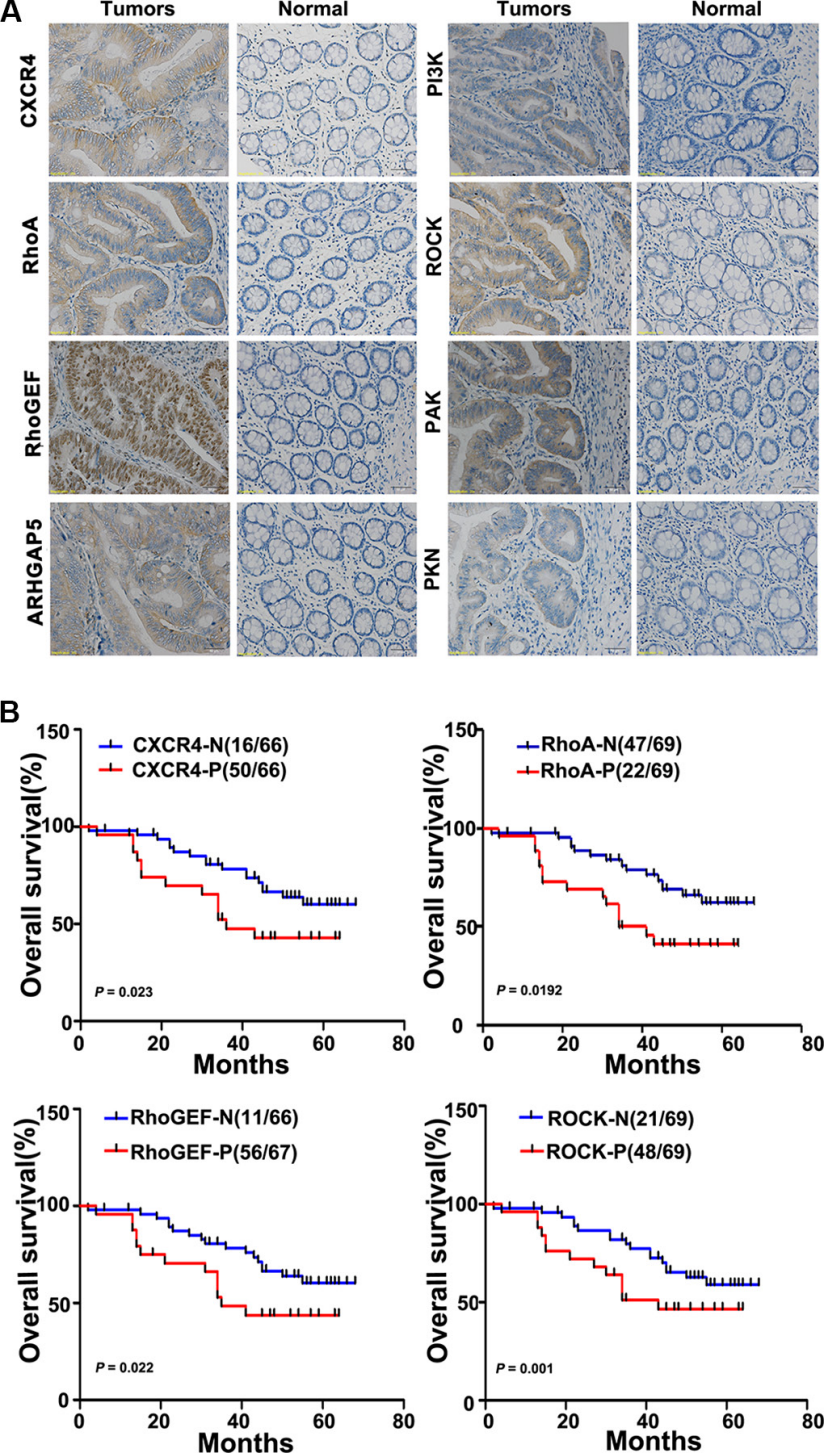
CXCR4 and RhoA signaling pathway components are upregulated in colon cancer tissues and inversely correlate with the overall survival of colon cancer patients (**A**) The relative levels of CXCR4 and the RhoA signaling pathway components RhoGEF, ARHGAP5, PI3K, ROCK, PAK, and PKN were assessed in colon cancer tissues and in paired normal colonic mucosa by immunohistochemical staining. Representative images are shown (200×). (**B**) Kaplan-Meier survival curves are shown for patients according to the expression of CXCR4, RhoA, RhoGEF, and ROCK in their colon cancer tissues. Colon cancer-associated death was significantly correlated with expression of CXCR4, RhoA, RhoGEF, and ROCK. Decreased patient overall survival was associated with higher expression of CXCR4 (upper left panel; *n* = 50/66; *p* = 0.023), RhoA (upper right panel; *n* = 47/69; *p* = 0.0192), RhoGEF (lower left panel; *n* = 56/67; *p* = 0.022), and ROCK (lower right panel; *n* = 48/69; *p* = 0.001). The red lines indicate patients with cancer tissues expressing CXCR4, RhoA, RhoGEF, and ROCK; blue lines indicate the patient tissues not expressing these proteins. In each graph, each ‘*N*’ implies negative expression, and each ‘*P*’ implies positive expression.

Previously, we found that miR-126 negatively targets CXCR4 and that it is reversely associated with RhoA activity [11, 12]. We thus examined the expression of CXCR4, RhoA, and key components of the RhoA signaling pathway in tissue microarrays. Immunohistochemical staining showed that CXCR4, RhoA, RhoGEF, ARHGAP5, PI3K, ROCK, PAK and PKN were significantly up-regulated in cancer tissues compared with normal tissues (Table 1, Figure 2A). We next analyzed the relationships between the levels of CXCR4, components of the RhoA signaling and clinicopathologic characteristics of colon cancer patients. The relationship between the expression levels of CXCR4 and RhoA signaling pathway components and the clinicopathological characteristics of colon cancer patients are summarized in Table 1. We found that higher levels of CXCR4, RhoA, RhoGEF, PI3K, and ROCK correlated positively with TNM stage (*p* < 0.05) and lymph node metastasis (*p* < 0.05) but not with age, gender, tumor size or tumor differentiation (Table 1). Kaplan-Meier plots were constructed to evaluate differences in survival based on the expression of CXCR4 and RhoA signaling pathway components (Figure 2B); decreased overall survival time was significantly associated with higher expression of CXCR4, RhoA, RhoGEF, and ROCK in the patient tissues. (*p* = 0.023; *p* = 0.0192; *p* = 0.022; *p* = 0.001, respectively; Figure 2B).

### Correlation among the expression of miR-126, CXCR4, and components of the RhoA signaling pathway

We further investigated the relationships among the expression levels of miR-126, CXCR4, and components of the RhoA signaling pathway based on *in situ* hybridization and immunohistochemistry. Spearman rank correlation analysis revealed that miR-126 expression was significantly and inversely correlated with CXCR4, RhoA, RhoGEF, and ROCK expression levels (*r* = − 0.391, *p* = 0.001; *r* = − 0.318, *p* = 0.005; *r* = − 0.252, *p* = 0.029; *r* = − 0.258, *p* = 0.026, respectively; Table 2); however, we found no obvious correlation between expression of miR-126 and that of PI3K, ARHGAP5, PAK, or PKN. Moreover, a significant positive correlation was found between expression of CXCR4 and of RhoA, RhoGEF, and ROCK, (*r* = 0.601, *p* = 0.000; *r* = 0.454, *p* = 0.000; *r* = 0.340, *p* = 0.005, respectively; Table 2), but not PI3K, ARHGAP5, PAK, or PKN based on immunohistochemical staining. These results indicated that miR-126 negatively correlated with CXCR4, RhoA, RhoGEF, and ROCK in human colon cancer.

**Table 2:** Correlation among the expression of miR-126, CXCR4, and components of the RhoA signaling pathway

Protein	miR-126 expression		*r*	*p*	CXCR4 expression		*r*	*p*
	(+)	(−)			(+)	(−)		
CXCR4 (+)	34	27	−0.391	0.001				
CXCR4 (−)	14	0						
RhoA (+)	28	24	−0.318	0.005	40	5	0.601	*P* <0.001
RhoA (−)	20	3			10	11		
RhoGEF (+)	37	26	−0.252	0.029	38	7	0.454	*P* <0.001
RhoGEF (−)	11	1			12	9		
ROCK (+)	29	23	−0.258	0.026	38	7	0.340	0.005
ROCK (−)	19	4			12	9		

### MiR-126 down-regulates the expression of CXCR4 and RhoA signaling pathway components in human colon cancer cells

CXCR4 is a direct target of miR-126 in human colon cancer cells [11], miR-126 is reversely associated with RhoA activity *in vitro* [12]. Given those results and our current findings, we hypothesized that miR-126 inhibits CXCR4 and the RhoA signaling pathway.

**Figure 3:**
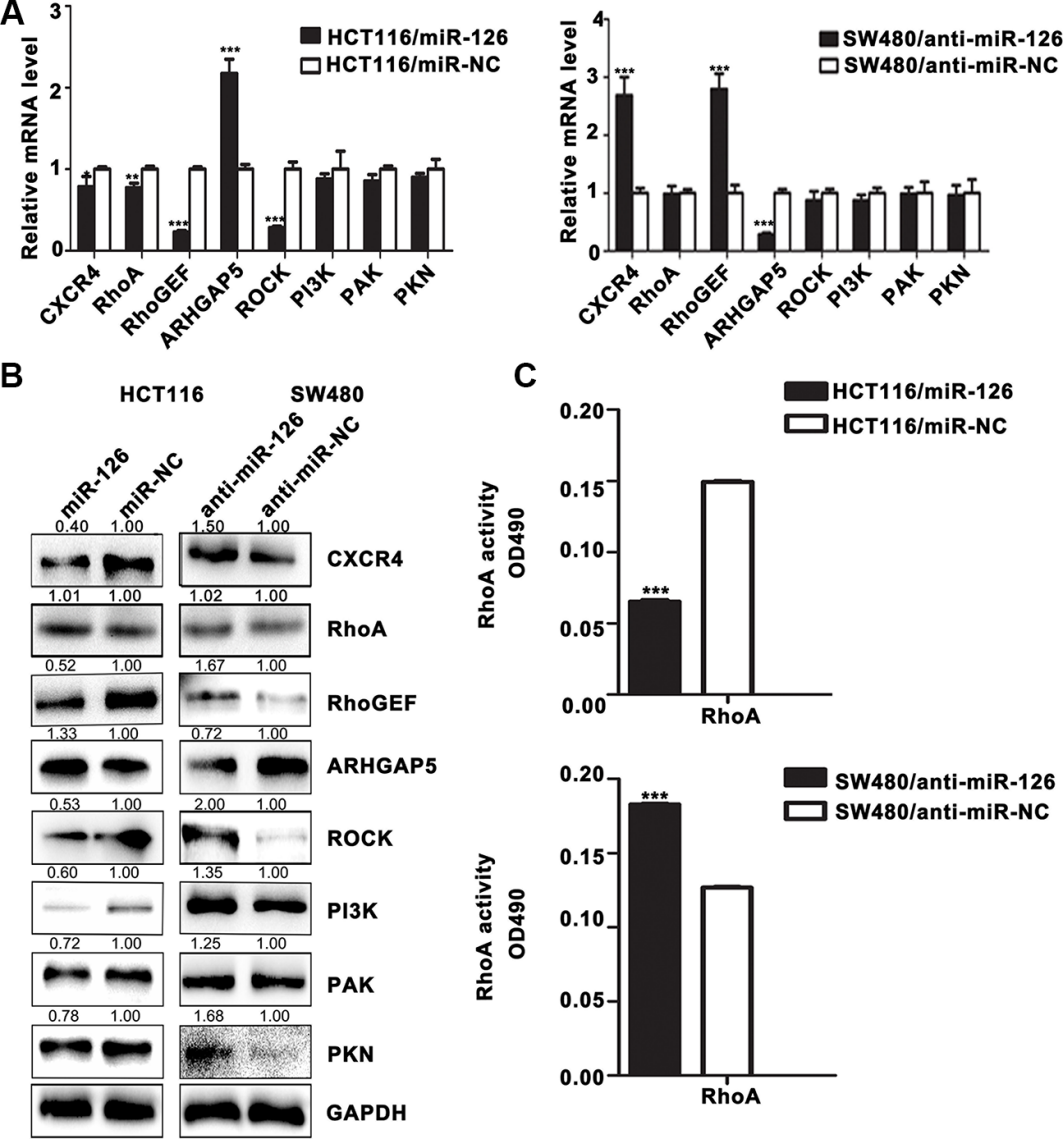
MiR-126 down-regulates the expression of CXCR4 and RhoA signaling pathway components in human colon cancer cells (**A**) The mRNA levels of CXCR4 and components of the RhoA signaling pathway were determined by qRT-PCR in transfected HCT116 and SW480 cells. GAPDH expression served as the internal control.*p < 0.05; **p < 0.01; ***p < 0.001. (**B**) Western Blot for the detection of CXCR4 and components of the RhoA signaling pathway in transfected HCT116 and SW480 cells. GAPDH served as the internal control. (**C**) RhoA G-LISA activation assays were used to measure RhoA activity in the transfected cells. ***p < 0.001.

As shown in Figure 3A, real-time PCR results revealed that overexpression of miR-126 in HCT116 cells decreased the expression of CXCR4, RhoA, RhoGEF, ROCK and up-regulated that of ARHGAP5 at the mRNA level compared with controls (*p* < 0.05, Figure 3A); and suppression of miR-126 in SW480 cells up-regulated the expression of CXCR4 and RhoGEF, and down-regulated that of ARHGAP5 compared with controls (*p* < 0.05, Figure 3A). Western Blot further revealed that miR-126 overexpression suppressed the expression of CXCR4, RhoGEF, ROCK, PI3K, PAK, and PKN, and up-regulated that of ARHGAP5 in HCT116 cells compared with controls; whereas blocking miR-126 expression resulted in the up-regulation of CXCR4, RhoGEF, PI3K, ROCK, PAK, and PKN expression and down-regulation that of ARHGAP5 in SW480 cells compared with controls (Figure 3B). The RhoA G-LISA activation assay showed that overexpression of miR-126 suppressed RhoA activity in HCT116 cells compared with controls (*p* < 0.05; Figure 3C), whereas silencing miR-126 significantly increased RhoA activity in SW480 cells compared with controls (*p* < 0.05; Figure 3D). And there was no significant difference in the expression of total RhoA (Figure 3B). Accordingly, our results revealed that miR-126 down-regulated the expression of CXCR4 and the critical components involved in RhoA signaling pathway in human colon cancer cells.

### CXCR4 is a functional mediator for miR-126 in colon cancer cells

**Figure 4:**
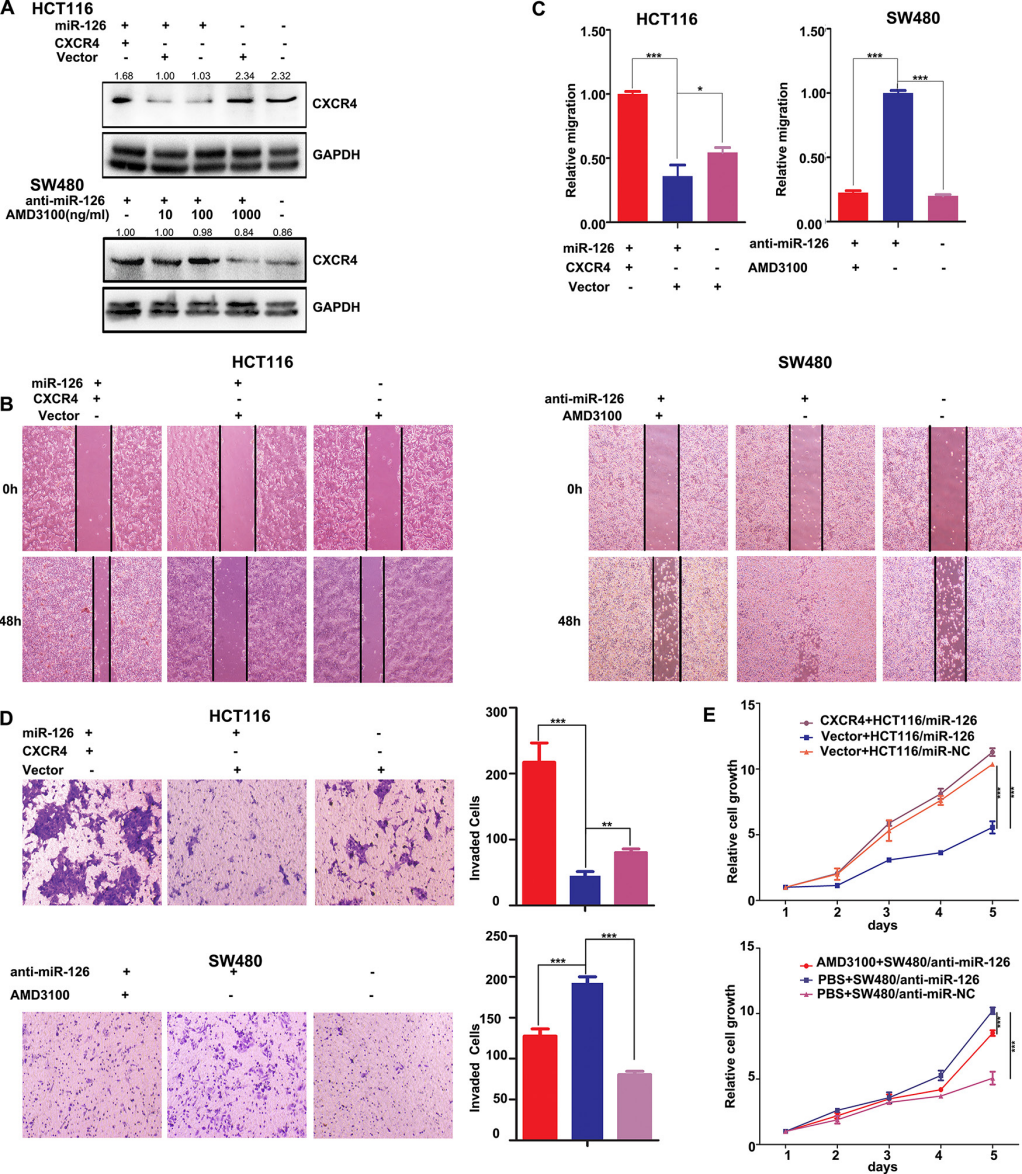
CXCR4 is a functional mediator for miR-126 in colon cancer cells (**A**) Western Blots were used to confirm CXCR4 expression. Transient transfection of HCT116/miR-126 cells with CXCR4 plasmids resulted in increased expression of CXCR4 compared with that cells transfected with unmodified vectors or not transfected (upper panels). AMD3100 was used to inhibit CXCR4 expression in SW480/anti-miR-126 cells (bottom panels). At a concentration of 1000 ng/ml AMD3100, CXCR4 expression was substantially reduced. (**B**, **C**) Wound-healing assays were used to measure cell migration capacity. Migration increased following transient transfection with the CXCR4 plasmids (left panels), but the migration of SW480/anti-miR-126 cells was inhibited after exposure to AMD3100 (right panels) *p < 0.05, ***p < 0.001. (**D**) Transwell assays were used to measure cell invasion capacity. The invasion capacity of HCT116/miR-126 cells increased following transient transfection with the CXCR4 plasmids (top). The red, blue, and purple bars denote CXCR4 + HCT116/miR-126, vector + HCT116/miR-126, and vector + HCT116/miR-NC, respectively. The invasion capacity of SW480/anti-miR-126 cells was reduced after exposure to AMD3100 (bottom). The red, blue, and purple bars denote AMD3100 + SW480/anti-miR-126, PBS + SW480/anti-miR-126, and PBS + SW480/anti-miR-NC, respectively. Panels on the left show stained cells that had invaded. Cells in five randomly selected areas were counted, and a statistical analysis was performed using SPSS 17.0. (**E**) MTT assays were used to measure cell proliferation. The proliferation of HCT116/miR-126 cells was increased after transient transfection with the CXCR4 plasmids (top panel). The proliferation of SW480/anti-miR-126 cells was reduced after exposure to AMD3100 (bottom panel). The data represent the mean ± SD of three replicates **p < 0.01, ***p < 0.001.

We next asked if the effects of miR-126 depend on CXCR4 level. To abrogate miR-126-mediated suppression of CXCR4, we transiently transfected CXCR4 plasmids into miR-126-overexpressing HCT116 cells, the expression of CXCR4 was confirmed by Western Blot (Figure 4A, top panels). And we also inhibited the expression of CXCR4 with AMD3100, a small-molecule antagonist of CXCR4, at concentrations of 10 ng/ml, 100 ng/ml, and 1000 ng/ml in miR-126-silienced SW480 cells. We found that AMD3100, particularly at 1000 ng/ml, potently inhibited CXCR4 expression (Figure 4A, bottom panels).

Functional assays showed that transfection with the CXCR4 plasmids in miR-126 overexpressing HCT116 cells significantly ameliorated the miR-126-induced suppression of colon cancer cell migration, invasion and proliferation, respectively (Figures 4B, 4C, 4D). Conversely, inhibition the expression of CXCR4 by AMD3100 in miR-126-silenced SW480 cells significantly countered the effects on migration, invasion and proliferation induced by silencing miR-126 expression (Figures 4B, 4C, 4D). These results suggested that CXCR4 reversed the miR-126-induced suppression of colon cancer cell migration, invasion and proliferation, potentially by acting as a mediator of miR-126 function.

### CXCR4 abolishes the effects of miR-126 on RhoA signaling pathway in colon cancer

**Figure 5:**
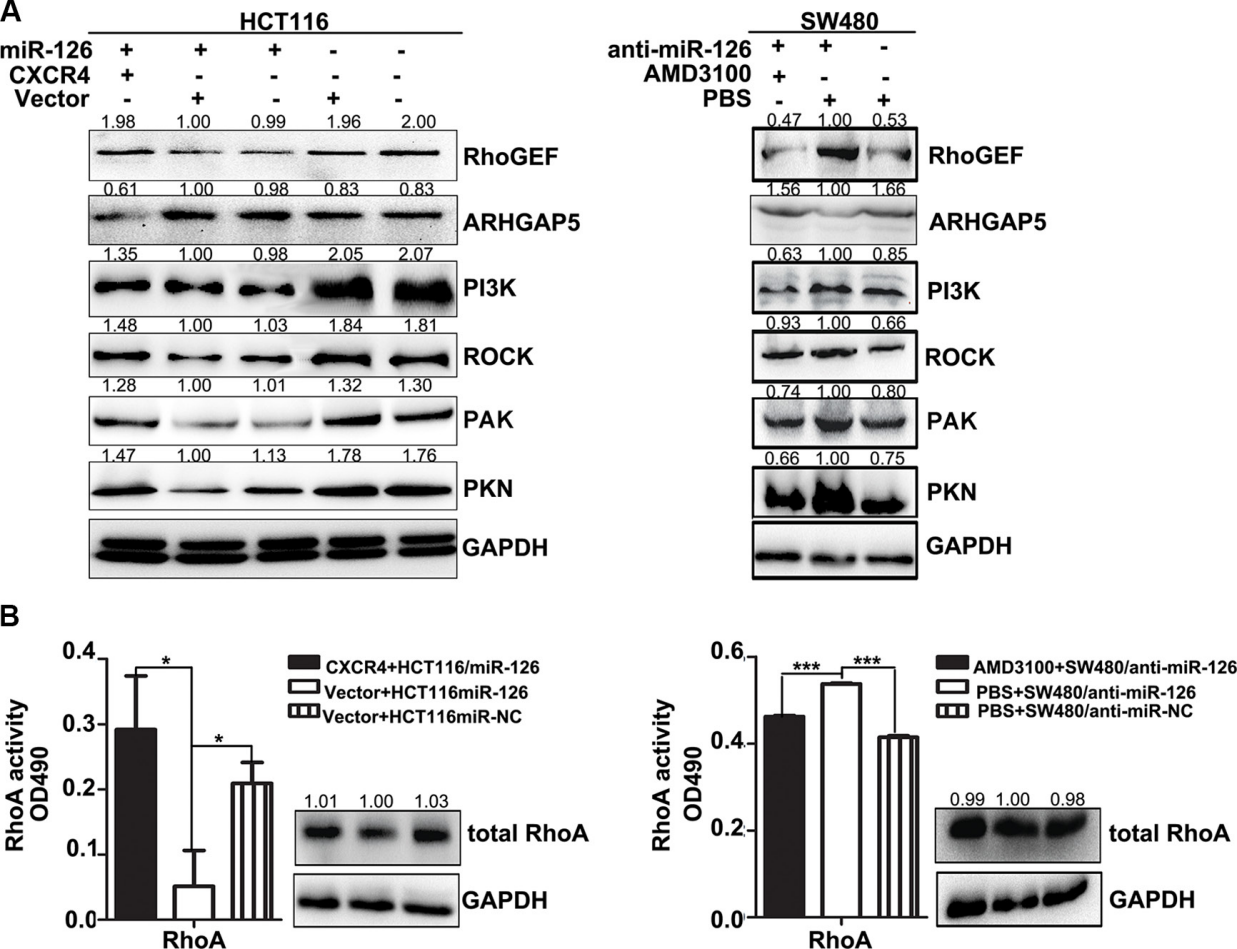
CXCR4 abolishes the effects of miR-126 on RhoA signaling pathway in colon cancer (**A**) Western blot analysis of the expression levels of components of the RhoA signaling pathway. Left panels show expression levels in HCT116/miR-126 cells after transient transfection with the CXCR4 plasmids. Right panels show expression levels in SW480/anti-miR-126 cells after exposure to AMD3100 (1000 ng/ml). (**B**) RhoA activation G-LISA assays were used to measure RhoA activity in HCT116/miR-126 cells transiently transfected with the CXCR4 plasmids (left panels) and in SW480/anti-miR-126 cells exposed to 1000 ng/ml AMD3100 (right panels). *p < 0.05, ***p < 0.001.

If CXCR4 indeed is a functional target of miR-126 in colon cancer cells, reintroduction of CXCR4 into miR-126 overexpressing HCT116 cells and inhibition of CXCR4 in miR-126-silenced SW480 cells should antagonize the effects of miR-126 on its target genes. Strikingly, we found that the expression of RhoGEF, PI3K, ROCK, PAK, PKN and the RhoA activity were up-regulated and that of ARHGAP5 was decreased after overexpression of CXCR4 in miR-126 overexpressing HCT116 cells. In contrast, the inhibition of CXCR4 in miR-126-silenced SW480 cells deceased the expression of CXCR4, RhoGEF, PI3K, ROCK, PAK, PKN and RhoA activity, up-regulated that of ARHGAP5 (Figures 5A, 5B). Accordingly, our results showed that CXCR4 can reverse the effects of miR-126 on RhoA signaling pathway in colon cancer.

### CXCR4 activates the RhoA signaling pathway via Gα_13_

**Figure 6:**
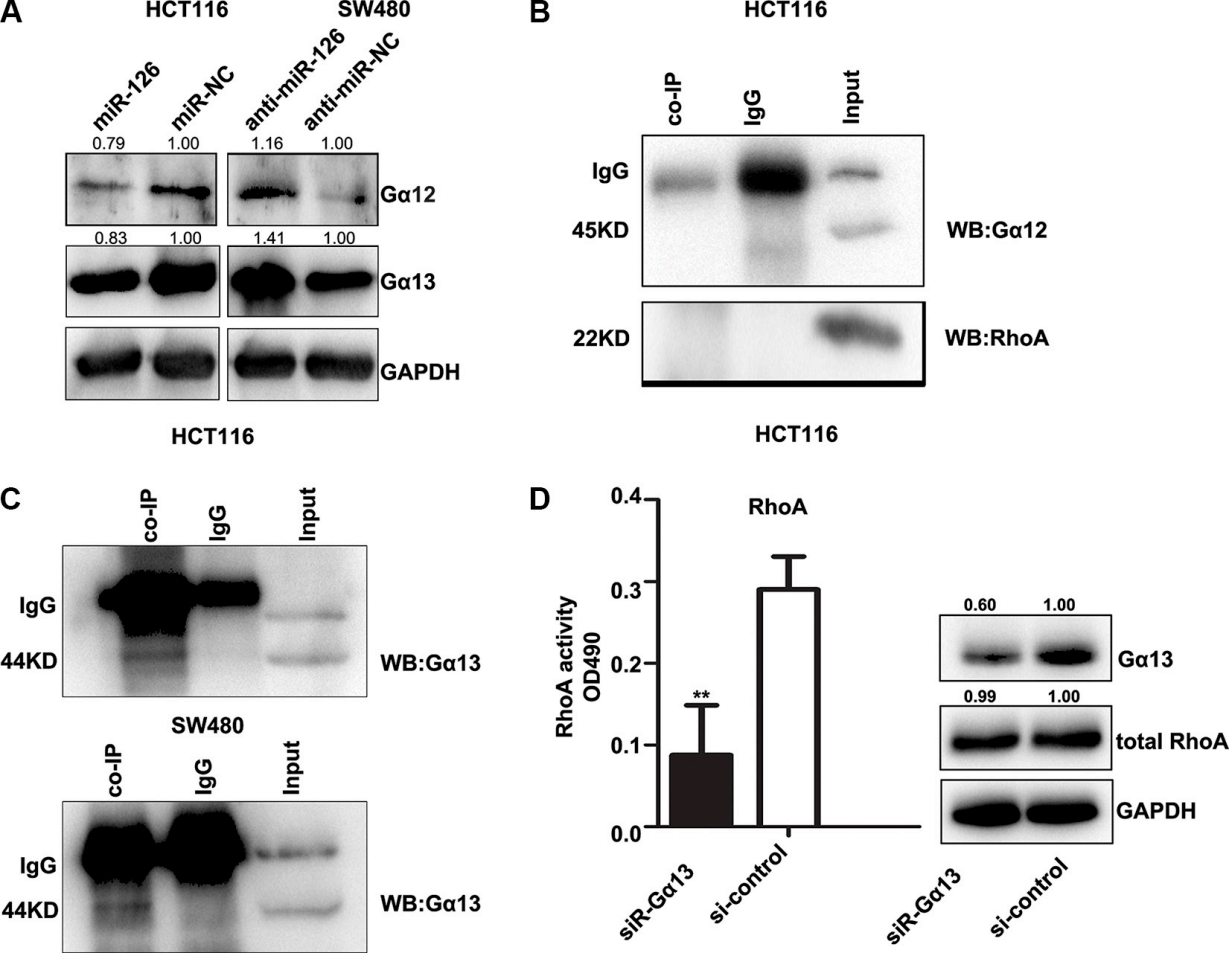
CXCR4 activates the RhoA signaling pathway via Gα_13_ (**A**) Western Blot showing the levels of Gα_12_ and Gα_13_ in transfected HCT116 and SW480 cells. GAPDH served as the internal control. (**B**, **C**) *In vitro* co-immunoprecipitation (co-IP) confirmed the interactions between CXCR4, RhoA, and the Gα_12_/Gα_13_ complex and revealed a complex composed of CXCR4 and Gα_13_. Total protein was extracted from untransfected HCT116 and SW480 cells. Total (input) and eluted (co-IP) proteins were subjected to Western Blot, which was performed with anti-Gα_12_, anti-RhoA, and anti-Gα_13_. (**D**) Western Blot was used to confirm the expression of Gα_13_ (right), and the G-LISA assay was used to measure RhoA activity in HCT116 cells after knocking down Gα_13_ expression with siRNA-Gα_13_ (left). The control group was exposed to the non-specific siRNA, **p < 0.01.

Gα _12_ and Gα _13_ may play essential role for CXCR4 activating Rho [29, 30]. Our previous studies showed that miR-126 inhibits the role of CXCR4. To study the relationship between miR-126 and Gα_12/13_, we assessed Gα_12_ and Gα_13_ expression in miR-126 overexpressing and miR-126 silenced colon cancer cells by Western Blot. Overexpression of miR-126 suppressed Gα_12_ and Gα_13_ level, whereas blocking miR-126 expression increased Gα_12_ and Gα_13_ level (Figure 6A).

To further elucidate the mechanism by which CXCR4 promotes Rho signaling, we examined if the Gα_12/13_/Rho signaling axis is required for CXCR4-induced Rho activation. Co-immunoprecipitation assays were performed in HCT116 and SW480 cells. Gα_13_ was detected in CXCR4 immune-complexes, but Gα_12_ and RhoA were not (Figures 6B, 6C), indicating that CXCR4 and Gα_13_ directly interacted in these cells.

To address whether Gα_13_ directly affects RhoA activity in our cell lines as mediated by CXCR4, we knocked down Gα_13_ expression using a siRNA, and Gα_13_ level was examined by Western Blot (Figure 6D, right panel). RhoA activity in HCT116 cells was significantly decreased after Gα_13_ knockdown, but no obvious change in total RhoA level was observed in either group. Taken together, our data suggested that CXCR4 is coupled to Gα_13_ and that Gα_13_ may mediate CXCR4 activation of RhoA signaling.

## DISCUSSION

Accumulating evidence suggests that miR-126 is a tumor suppressor. And miR-126 is markedly down-regulated in various tumor tissues, including lung cancer [9, 10], breast cancer [31], prostate cancer [32], gastric cancer [7, 8] and oral squamous cell carcinoma [33]. Our previous studies [11, 12] and those of others [34, 35] found that miR-126 suppresses colon cancer cell proliferation and invasion *in vitro*, but the potential role and mechanisms of miR-126 in colon cancer progression had not been fully elucidated. We have now expanded our studies concerning the roles and the possible mechanisms of miR-126 in colon cancer *in vitro* and *in vivo*.

Our findings with mouse xenografts revealed that overexpression of miR-126 decreased tumorigenicity and metastasis capacity of HCT116 cells *in vivo*, whereas knockdown of miR-126 expression in SW480 induced tumorigenicity and metastasis. Consistent with these *in vivo* and previous *in vitro* results, the tissue microarray experiments showed that the expression of miR-126 is relatively down-regulated in human colon cancer and that reduced miR-126 expression is associated with TNM stage, lymph node metastasis, and most importantly, the lower survival rate for colon cancer patients. Combined with our previous studies [11–13], these results indicated that miR- 126 suppresses colon cancer progression *in vivo* and *in vitro* and in human colon cancer tissues.

However, the precise molecular mechanisms by which miR-126 inhibits colon cancer proliferation and metastasis remain unknown. Our previous studies preliminarily indicated that CXCR4 and RhoA/ROCK signaling pathway may be involved in the mechanisms of miR-126. And CXCR4 is negatively targeted by miR-126. Based on our previous studies, we used tissue microarrays to show that CXCR4 and components of the RhoA signaling pathway were up-regulated in colon cancer tissue compared with those in adjacent normal mucosa tissues. And they were significantly correlated with TNM stages, lymph node metastasis and poor clinical outcome. MiR-126 negatively correlated with CXCR4, RhoA, RhoGEF and ROCK in human colon cancer. These results implied that miR-126/CXCR4/RhoA signaling is possibly a key pathway that regulates the malignant phenotype of colon cancer. Consistent with the findings of colon cancer tissue microarray, we also found that miR-126 significantly suppressed the expression of CXCR4, the activity of RhoA, and the expression of RhoGEF, ROCK, PI3K, PAK, PKN and up-regulated the expression of ARHGAP5 in colon cancer cells. Our studies *in vitro* and *in vivo* implied that miR-126 is a negative regulator of CXCR4 and RhoA signaling pathway.

CXCR4 is believed to be a key factor in the cross-talk between cancer cells and their microenvironment, which makes CXCR4 a very promising prognostic biomarker and target for cancer therapy [36]. Wang et al. [37] found that silencing CXCR4 blocks the progression of ovarian cancer. In addition, it is reported that blocking the CXCR4/mTOR signalling pathway induces the anti-metastatic properties and autophagic cell death in peritoneal disseminated gastric cancer cells [38]. Wang and colleagues [39] reported that silencing of CXCR4 by RNA interference inhibits cell proliferation and metastasis of human renal cancer cells.

In our studies, we demonstrated that restoration of CXCR4 could significantly reverse the effect of miR-126 on the migration, proliferation and invasion of colon cancer cells, and abolish the effects of miR-126 on RhoA signaling. These results verified the central role of CXCR4 in miR-126-mediated suppression of colon cancer malignancy and CXCR4 is essential for miR-126 inhibiting RhoA signaling pathway.

It is reported that CXCR4 promotes cell migration and invasion via the CXCR4/Gα_13_/Rho signaling axis in breast cancer [29]. Gα_12_ and Gα_13_ (which together define the Gα_12/13_ protein family) can activate RhoA [40, 41]. Gα_13_ promotes Rho activation by binding to the RGS (Regulator of G-protein Signaling) domain in Rho GEFs [42]. In our results, we found that CXCR4 is directly coupled to Gα_13_ but not to RhoA or Gα_12_, and interference with Gα_13_ expression decreased RhoA activity. These data suggested that Gα_13_ subunit is essential for CXCR4 activating RhoA signaling, and CXCR4/Gα_13_/Rho signaling axis may promote the malignant phenotype of colon cancer. Further research is needed to confirm these results.

**Figure 7:**
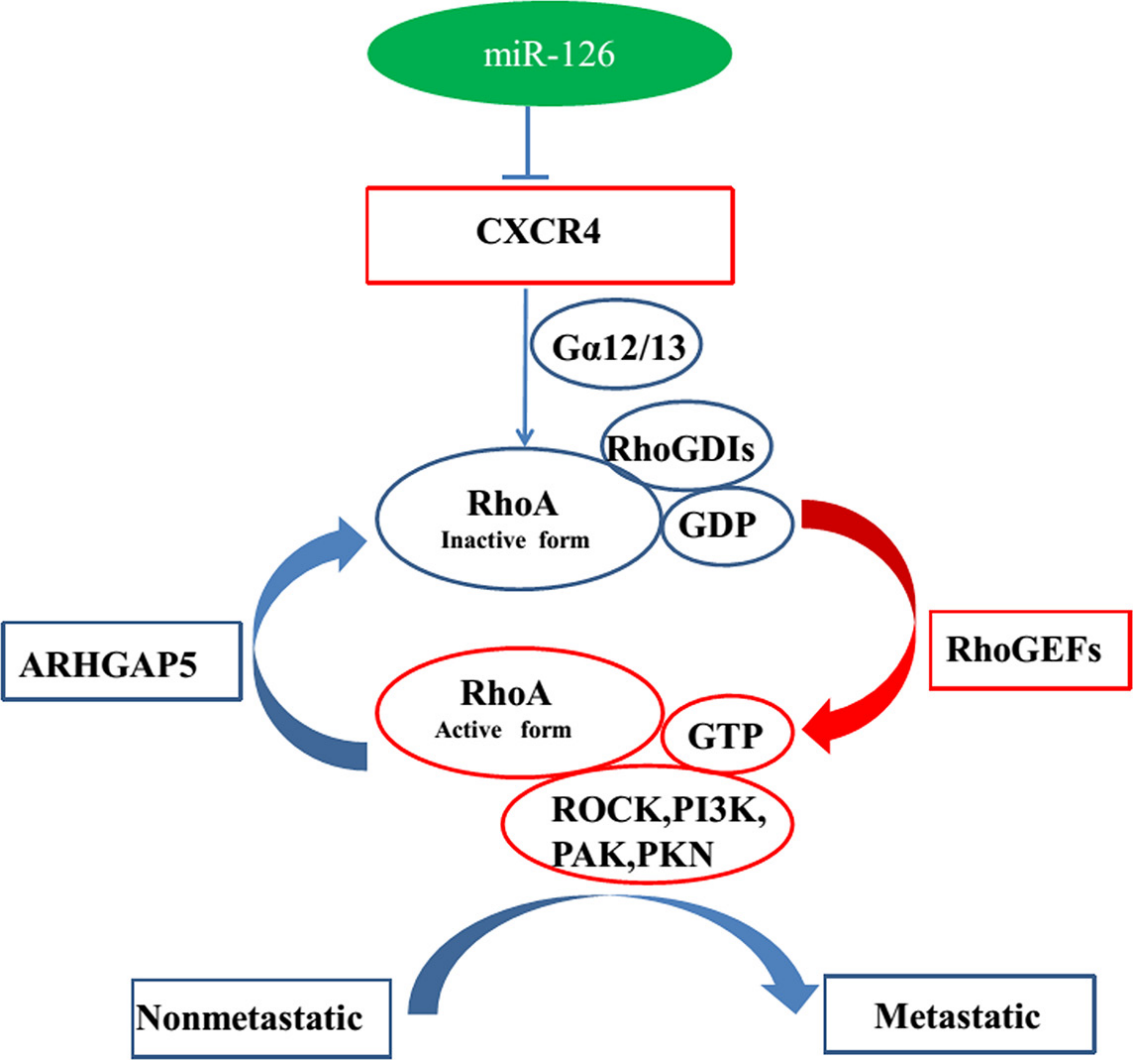
Model for miR-126-mediated inhibition of cell proliferation and migration via its regulation of the CXCR4 and RhoA signaling pathways Rho GDP-dissociation inhibitors (RhoGDIs) are important regulators of the Rho family of small GTPases. RhoGDIs can prevent RhoA activation by inhibiting GDP dissociation from RhoA. RhoGEFs activate RhoA, and ARHGAP5 can negatively regulate RhoA. PAK, PKN, ROCK, and PI3K are downstream effectors of RhoA signaling. MiR-126 inhibits cell proliferation and migration via its regulation of the CXCR4/Gα_13_/RhoA signaling axis in colon cancer pathology.

Liu et al. [35] found that miR-126 functions as a tumor suppressor in colorectal cancer cells by targeting CXCR4 via the AKT and ERK1/2 signaling pathways. In the present study, we discovered the novel regulation pathway of miR-126, which for the first time validated the mechanism of miR-126 acts as tumor suppressor, via regulation of the CXCR4 and RhoA signaling pathway both *in vitro* and *in vivo* (Figure 7). These data extended the regulation network of miR-126 and membrane receptor CXCR4 which provide a rationale for future application of miR-126/CXCR4/RhoA based diagnostic marker and targeted therapy.

## MATERIALS AND METHODS

### Cell lines and cell culture

The colon cancer cell line HCT116 (with relatively high invasive potential [43] and low miR-126 expression level [12]), and the colon cancer cell line SW480 (with relatively low invasive potential [44] and high miR-126 expression level [12]), were obtained from the Cancer Research Institute of the Central South University, China. HCT116 cells were cultured in DMEM with high glucose and sodium pyruvate, SW480 cells were cultured in 1640 medium. The medium were supplemented with 10% (v/v) fetal calf serum (FBS), 100 U/ml penicillin and 100 mg/ml streptomycin (all from GIBCO). And the cells were incubated at 37°C in a humidified atmosphere of 5% CO_2_ in air.

### Plasmids and reagents

The plasmid (NM_003467) for the construction of the human CXCR4 vector, which was also used in unmodified form as the negative control vector, was purchased from Genechem. The siRNA-Gα_13_ was used to target human Gα_13_, and the non-silencing siRNA (si-control; GenePharma) served as the control. AMD3100 (GenePharma), used to inhibit CXCR4, was dissolved in sterile PBS as a 1mg/ml stock solution and then diluted into the culture medium to a final concentration of 10, 100 or 1000 ng/ml. Rabbit polyclonal primary antibodies against CXCR4 (sc-9046), Gα_12_ (sc-409), and Gα_13_ (sc-410), mouse monoclonal antibodies against GAPDH (sc-166545), RhoA (sc-418), PAK (sc-166174), PKN (sc-136037), PI3K (sc-166365), ROCK (sc-17794), and RhoGEF (sc-166301), and the horseradish peroxidase–conjugated secondary antibodies against rabbit-IgG (sc-2004) and mouse-IgG (sc-2005) were purchased from Santa Cruz Biotechnology. The mouse monoclonal antibody against GAPDH (AB-2302) was purchased from Millipore (MA). The primary rabbit polyclonal antibody against ARHGAP5 was purchased from Sigma-Aldrich.

### Generation of stable transformants

The miR-126 lentivirus expression and knockdown systems were purchased from SBO Medical Biotechnology. In brief, a concentrated solution of virus was made via ultracentrifugation and purification, and the titer of virus was determined. Subsequently, the lentiviral systems were used to infect HCT116 or SW480 cells. Briefly, the cells were cultured in 6-well plates. When the culture reached 80% confluency, the concentrated lentivirus was added to the culture dishes. Stable transfected cells were sorted using fluorescence-activated cell sorting and maintained and selected in the presence of 1 ug/ml puromycin (Sigma-Aldrich). qRT-PCR was performed to determine the level of miR-126 expression.

### Human colon cancer tissue microarrays

Nine human colon cancer tissue microarrays were purchased from Shanghai Outdo Biotech. (Serial numbers OD-CT-Dg Co 101–109). Each microarray contained human colon cancer tissue and adjacent, normal colonic mucosa tissues that were collected at the time of surgical resection from 75 colon cancer patients between 2007 and 2008 in Shanghai, China. We excluded tissues of patients who had received neoadjuvant chemotherapy and/or radiotherapy or had unresectable colon cancers. The demographic and clinicopathological data for the patients are listed in Supplementary Table S1. The colon cancer tissue microarrays were then ready for *in situ* hybridization and immunohistochemistry. Note that some samples were lost during experimentation, thereby reducing the number of available cancer tissue samples to 609 pots and the number of normal tissue samples to 581 pots.

### Gene transfection

Upon reaching 70–80% confluency, transfected cultured HCT116 cells and SW480 cells were trypsinized, counted, and 7× 10^6^ cells/well were seeded into the wells of 6-well plates. MiR-126 overexpressing HCT116 cells and their control cells were transfected with the CXCR4 or unmodified CXCR4 vector with the use of Lipofectamine 2000. MiR-126 silenced SW480 cells and their controls were exposed to AMD3100 or PBS. The HCT116 cell lines were transfected with siRNA specific for Gα_13_ or si-control with the use Lipofectamine 2000. At 48 h post-transfection, Western Blot was used to verify transfection efficiency, and experiments were performed as described below.

### MTT assay

Cell proliferation was assessed using MTT assay. Briefly, 2000 cells/well were seeded in 96-well plates. On days 1–5, 20 μl of the MTT solution (Sigma-Aldrich, 5 mg/ml) was added to each well. After incubation at 37°C for 4 h, supernatants were removed, and 150 μl dimethylsulfoxide (Sigma**-**Aldrich) was added to each well. The plates were shaken for 10 min to dissolve the MTT formazan crystals. The OD_490_ of each dimethylsulfoxide sample was then measured. Data were from three separate experiments with three replications.

### Tumor cell wound-healing assay

Cells (5 × 10^6^ per well) were seeded in 6-well plates and cultured in medium containing 10% (v/v) FBS. When the cells reached 85% confluency, wounds were produced by scraping the cells with a 10 μl pipette tip. Cells were then cultured in medium containing 2% (v/v) FBS, and their migration at the wound site was documented using an inverted microscope at 0 and 48 h. The wound gaps were photographed.

### Matrigel invasion assay

Before cell seeding, 24-well Transwell plates (8-μm pores; Corning) were pre-coated with 10 μl Matrigel Matrix (BD Biosciences). Cells (1×10^5^cells/well) were seeded onto Transwell membrane inserts in serum-free medium. Then, medium containing 15% (v/v) FBS was added into the lower chamber. After incubation for 24 to 48 h at 37°C, the cells on the lower membrane surface were fixed in 4% (w/v) paraformaldehyde for 15 min and allowed to air dry at room temperature. Then the invasive cells were stained with 2% (w/v) crystal violet for 5 min, and the stained cells were counted under an inverted microscope. Three independent fields in each well were photographed, and cells in five random fields in each photograph were counted at 100× magnification.

### RNA isolation and quantitative Real-time PCR

Total RNA was extracted from cell lines using Trizol reagent (Invitrogen) and then treated with DNase (Roche Diagnostics) to eliminate contaminating DNA. cDNA was synthesized from 2 μg of total RNA by reverse transcription reagents (Fermentas). Expression of the mRNAs was evaluated using SYBR green qRT-PCR (TaKaRa) in accordance with the standard protocol. GAPDH was amplified in parallel as an internal control for assay of the mRNAs. The individual miRNA qRT-PCR Quantitation kit was purchased from Genepharma, and U6 small nuclear RNA was used as an endogenous control for assay of miRNA expression levels. Expression of each gene was quantified by measuring cycle threshold (Ct) values and normalized using the 2^−ΔΔCt^ method relative to U6 small nuclear RNA or GAPDH mRNA [45]. The data are representative of the means of three times. RT-PCR primers are listed in Supplementary Table S2.

### Western blot

In Western Blot analysis, total proteins were extracted from the cultured cells using RIPA buffer with both phosphatase and protease inhibitors (Roche, IN). Equal aliquots of 50 μg total proteins were separated by a SDS-PAGE (10% acrylamide). Then they were transferred to a PVDF membrane (Merck Millipore, Germany). Membranes were blocked in TBST containing 5% skim milk and then incubated with primary antibodies overnight at 4°C followed by secondary antibodies for 1 h at 37°C. Finally, ECL detection system (Merck Millipore, Germany) was used for signal detection.

### Protein co-immunoprecipitation

Proteins were extracted from whole cells, and equivalent amounts of protein lysates were incubated with anti-CXCR4 (diluted 1:200) anti-IgG (diluted 1:200; catalog no. 2729S, Millipore) at 4°C overnight and then incubated with 40 μl of protein A/G agarose beads (Santa Cruz Biotechnology) at 4°C for 4 h. Immunoprecipitates were washed three times with 1 ml cold PBS. Immunoprecipitated proteins were eluted with SDS-PAGE sample buffer and then subjected to SDS-PAGE (10% acrylamide) and Western Blot with anti-Gα_12_, anti-Gα_13_, and anti-RhoA.

### G-LISA RhoA-activation assay

The G-LISA RhoA-activation assay was carried out using the reagents of the RhoA G-LISA Activation Assay kit (Cytoskeleton Inc.). Briefly, cells were grown to 80% confluency and then serum starved overnight. On the following day, cells were lysed, and the lysates were quick frozen in liquid nitrogen and stored at −80°C until use. Supernatants (60 μl) were used for protein concentration determination using the Precision Red Advanced Protein Assay supplied with the kit. For quantitative detection of active RhoA, 1 mg/ml protein was used and absorbance was read at 490 nm using a microplate ELISA reader. Supernatants were also used for the Western Blot for total RhoA.

### *In situ* hybridization and immunohistochemical staining

*In situ* hybridization was used to detect miR-126 expression in the human colon cancer tissue microarray with a Hsa-miR-126 probe (/5DigN/GCATTATTACTCACGGTACGA/3Dig_N/; Exiqon). MiR-126 by ISH was performed utilizing an enhanced sensitive ISH detection kit (Boster Inc.).

Deparaffinized sections were incubated with a primary antibody at 4°C overnight followed by incubation with biotin-linked goat anti-rabbit IgG (UltraSensitive S-P kit, Maixin Biotechnology). Antibodies were those used for Western Blot. Omission of each primary antibody served as the corresponding negative control.

Finally, the sections were observed and imaged under a microscope (OLYMPUS BX-51, Japan) by two independent pathologists blinded to clinicopathologic features and clinical courses. A semi-quantitative scoring criterion for *in situ* hybridization and immunohistochemistry was used, in which both staining intensity and positive areas were recorded. Intensity was scored as 0 (no staining), 1 (weak staining), 2 (moderate staining), and 3 (strong staining). The percentage of positive tumor cells was set as follows: 0 (< 5%), 1 (5– 25%), 2 (26–50%), 3 (51–75%) and 4 (> 75%). The sum of intensity and percentage counts was used as the final score. And it was scored as 0(−),1–2 (+), 3–5 (++), 6–7 (+++). And the final score = 0 was defined as negative expression, otherwise was defined as positive expression.

### Tumorigenicity and metastasis assays in nude mice

Forty-eight male BALB/c nude mice aged 4 weeks were used for the tumorigenicity and metastasis assays. All animal studies and experimental protocols were approved by the Ethics Committee for Animal Experimentation of Central South University. MiR-126 overexpressing HCT116 cells, miR-126 silenced SW480 cells and their respective controls (5 × 10^6^ cells) were implanted into the right flank of each mouse (6 mice/group). Tumor growth was monitored starting 1 week after injection and every 3 d thereafter by measuring tumor volume using a caliper, which was calculated as (longest length) × (shortest length)^2^ × 0.52 [46]. Mice were euthanized 31 d after injection, and tumors were excised.

MiR-126 overexpressing HCT116 cells, miR-126 silenced SW480 cells and their respective controls (10^6^ cells) were injected into the lateral tail vein of each mouse (6 mice/group). Mice were euthanized 50 d later, and their lungs were removed to determine the micrometastases.

### Statistical analysis

The statistical significance of differences between groups was assessed using the Student's *t*-test in SPSS 17.0 and GraphPad Prism 5. ANOVA was used for comparison of tumor volumes between groups. Survival data were analyzed using Kaplan-Meier analyses. The χ^2^ test was used to evaluate the relationship between the expression of miR-126, CXCR4, and RhoA signaling components and clinicopathological characteristics. Spearman's rank test was used to analyze the correlations among miR-126, CXCR4 and RhoA signaling components. All values are expressed as the means ± SD. *P* values less than 0.05 were considered significant.

## SUPPLEMENTARY MATERIALS FIGURE AND TABLES


