# Supplementary material for: MicroRNA-126 inhibits colon cancer cell proliferation and invasion by targeting the chemokine (C-X-C motif) receptor 4 and Ras homolog gene family, member A, signaling pathway
Source: Oncotarget. 2016 Aug 10;7(37):60230–44. doi: 10.18632/oncotarget.11176 (PMC5312381; doi:10.18632/oncotarget.11176)
Supplement: Supplementary file 1 [file oncotarget-07-60230-s001.pdf]

MicroRNA-126 inhibits colon cancer cell proliferation and invasion by targeting the chemokine (C-X-C motif) receptor 4 and Ras homolog gene family, member A, signaling pathway

Supplementary Materials

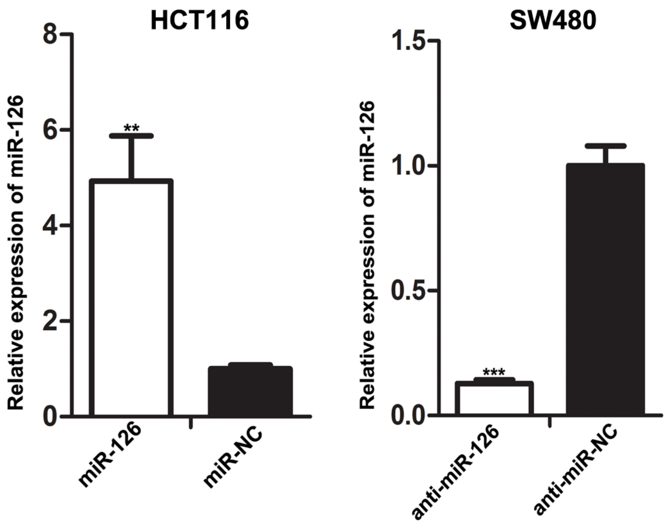

Supplementary Figure S1: Establishment of miR-126 overexpression HCT116 cell line and miR-126 silenced SW480 cell line. Relative expression of miR-126 in HCT116/miR-126 cells (overexpressing miR-126), HCT116/miR-NC cells (control), SW480/anti-miR-126 cells (suppressed miR-126 expression), and SW480/anti-miR-NC cells (control) was examined by qRT-PCR. U6 small nuclear RNA served as the internal qRT-PCR control. $^{**}p < 0.01$; $^{***}p < 0.001$.

Supplementary Table S1: Demographic and clinicopathological data

Parameters	Number
**Age (years)**	
< 60	26 (34.7%)
> 60	49 (65.3%)
**Gender**	
Male	38 (50.7%)
Female	37 (49.3%)
**Tumor differentiation**	
Well	10 (13.3%)
Moderate	53 (70.7%)
Poor	12 (16%)
**TNM stage**	
I	12 (16%)
II	23 (30.7%)
III	31 (41.3%)
IV	9 (12%)
**Lymph node metastasis**	
Yes	37 (49.3%)
No	38 (50.7%)

Supplementary Table S2: PCR primer list

Gene product	Forward /reverse primer	Sequence
CXCR4 (145 bp)	FORWARD	5′-GGAGGGGATCAGTATATACA-3′
	REVERSE	5′-GAAGATGATGGAGTAGATGG-3′
RhoA (196 bp)	FORWARD	5′-ATTCGTTGCCTGAGCAATGG-3′
	REVERSE	5′-TGTGTCCCACAAAGCCAACT-3′
ROCK (292 bp)	FORWARD	5′-ATGTGACTGGTGGTCGGTTG-3′
	REVERSE	5′-AACTGGTGCTACAGTGTCTCG-3′
PKN (188 bp)	FORWARD	5′-CATGAGAAGGCTGCTTCGGA-3′
	REVERSE	5′-ACTCCTCGTCGAAGTTGCTG-3′
PI3K (117 bp)	FORWARD	5′-TTGTTCCAATCCCAGGTGGA-3′
	REVERSE	5′-TTAGCACCCTTTCGGCCTTT-3′
PAK (231 bp)	FORWARD	5′-AGCTGCTACAGGTGAGAAAACT-3′
	REVERSE	5′-AGAGGGCATCAGGAGTTGGA-3′
RhoGEF (270 bp)	FORWARD	5′-GCGCGGACACCAGCC-3′
	REVERSE	5′-GCAGACAGCAAAGCAGGG-3′
GAPDH (238 bp)	FORWARD	5′-CGACCACTTTGTCAAGCTCA-3′
	REVERSE	5′-AGGGGTCTACATGGCAACTG-3′
ARHGAP5 (164 bp)	FORWARD	5′-CATCTGTTTTTTGGCCAACCT-3′
	REVERSE	5′-GTGGAGGAGCCACAATGTTT-3′
